# THE METHODOLOGICAL RELEVANCE OF MASS OBSERVATION DATA

**DOI:** 10.1093/geroni/igz038.2778

**Published:** 2019-11-08

**Authors:** Charlotte L Eost-Telling, Paul Kingston, Louise Taylor, Jan Bailey

**Affiliations:** University of Chester, Chester, England, United Kingdom

## Abstract

The Mass Observation Project, established in 1937, documents the lives of ordinary people living in the UK, and explores a wide range of social issues. The Project distributes a set of written questions (“Directives”) to a panel of 500 members of the British public (“Observers”) three times each year; “Observers” respond in writing. From the initial commissioning of a “Directive” to data becoming available for analysis takes between four to six months. This approach offers researchers an opportunity to capture in-depth qualitative data from individuals with a range of demographic backgrounds who live across the UK. As there are no word limits on “Observers’” responses and they remain anonymous, a “Directive” often yields rich, high-quality data. Additionally, compared with alternative methods of collecting large volumes of qualitative data from a heterogeneous population, commissioning a “Directive” is cost-effective in terms of time and resource.

